# Supplementary material for: SARS-CoV-2 antibody persistence after five and twelve months: A cohort study from South-Eastern Norway
Source: PLoS One. 2022 Aug 10;17(8):e0264667. doi: 10.1371/journal.pone.0264667 (PMC9365168; doi:10.1371/journal.pone.0264667)
Supplement: S1 Table — (DOCX) [file pone.0264667.s001.docx]

**S1. Table.** **Questions from the questionnaire concerning demographic data, hospitalisation, comorbidities, symptoms and fatigue**

| Income (NOK, one NOK= 0.096 Euro) | What is the total gross income in your household | <500 000 | > 500 000 | >1 million |
| --- | --- | --- | --- | --- |
| Education | What is your highest education? | Primary & secondary school | High school & certificate | University < or > than 4 years |
| Smoking habits | Do you smoke? | Non-smoker? | Past smoker? | Occasionally and daily? |
| Asthma | Has a physician ever diagnosed you with asthma? |  |  |  |
| Chronic obstructive pulmonary disease (COPD) | Has a physician ever diagnosed you with COPD? |  |  |  |
| Do you have the following disease? | Other lung disease? |  |  |  |
|  | Cancer? |  |  |  |
|  | Heart disease? |  |  |  |
|  | Diabetes? |  |  |  |
|  | Hypertension? |  |  |  |
|  | Musculoskeletal disease? |  |  |  |
|  | Any other disease? |  |  |  |
|  | No disease? |  |  |  |
| Pollen allergy | Do you have pollen allergy? |  |  |  |
| Hospitalisation | Have you been hospitalized for COVID-19? |  |  |  |
| Symptoms | Have you been ill with symptoms from airways, feeling ill or having fever when you had COVID-19? |  |  |  |
| Did you have symptoms? | Cough? | Runny nose? Stuffy nose? | Sore throat? | Pain in swallowing? |
|  | Dyspnoe? | Headache? | Fever? | Fever with chills or sweating? |
|  | Abdominal pain, nausea or diarrhea? | Impaired sense of smell and taste? | Myalgia? | Dizziness? |
| Fatigue: Have you had fatigue you relate to time after PCR test was taken? | 0=no fatigue | 1= mild fatigue | 2=moderate fatigue | 3=serious fatigue |
