# Supplementary material for: SARS-CoV-2 antibody persistence after five and twelve months: A cohort study from South-Eastern Norway
Source: PLoS One. 2022 Aug 10;17(8):e0264667. doi: 10.1371/journal.pone.0264667 (PMC9365168; doi:10.1371/journal.pone.0264667)
Supplement: S2 Table — The median time between positive PCR and T1 antibody measurement was 127 days (91–153) and between positive PCR and T2 antibody 310 days (291–329). Among the PCR negative participants, the median time between PCR test and T1 total antibody was 200 days (114–217). (DOCX) [file pone.0264667.s002.docx]

**S2 Table 2.** **The median time between positive PCR and antibody measurements.**

The median time between positive PCR and T1 antibody measurement was 127 days (91-153) and between positive PCR and T2 antibody 310 days (291-329). Among the PCR negative participants, the median time between PCR test and T1 total antibody was 200 days (114-217).

| **Time in days** | **Median (IQR), range n** | **Median (IQR), range n** | **Median (IQR), range n** |
| --- | --- | --- | --- |
| Time from PCR test to T1 antibody | 154.0 (104.0-207.0) | 127.0 (91.0-153.0) | 200.0 (114.8-217.0) |
|  | 19-275, 1094 | 19-234, 391 | 23-275, 703 |
| Time from PCR test to T2 antibody | 310.0 (291.0-329.0) | 310.0 (291.5-328.8) | 264.0* |
|  | 192-421, 215 | 192-421, 212 | 261-391, 3 |
| Time from T1 antibody test to T2 | 167.0 (161.0-182.0) | 167.0 (161.0-181.0) | 203.0* |
|  | 113-273, 215 | 113-273, 212 | 181-210, 3 |

| ^a^ Lower and upper quartiles were not given due to too few observations. |  |  | |
| --- | --- | --- | --- |
|  | | |  |
